# Injection of resistin into the paraventricular nucleus produces a cardiovascular response that may be mediated by glutamatergic transmission in the rostral ventrolateral medulla

**DOI:** 10.22038/IJBMS.2023.69324.15110

**Published:** 2024

**Authors:** Abolfazl Akbari, Gholamali Jelodar, Saeid Hosseinzadeh

**Affiliations:** 1 Department of Basic Sciences, School of Veterinary Medicine, Shiraz University, Shiraz, Iran; 2 Department of Food Hygiene and Public Health, School of Veterinary Medicine, Shiraz University, Shiraz, Iran

**Keywords:** Arterial pressure, Glutamatergic transmission, Heart rate, Paraventricular, Hypothalamic nucleus, Resistin

## Abstract

**Objective(s):**

High levels of resistin are associated with metabolic diseases and their complications, including hypertension. The paraventricular nucleus (PVN) is also involved in metabolic disorders and cardiovascular diseases, such as hypertension. Therefore, this study aimed to study cardiovascular (CV) responses evoked by the injection of resistin into the lateral ventricle (LV) and PVN and determine the mechanism of these responses in the rostral ventrolateral medulla (RVLM).

**Materials and Methods:**

Arterial pressure (AP) and heart rate (HR) were evaluated in urethane-anesthetized male rats (1.4 g/kg intraperitoneally) before and after all injections. This study was carried out in two stages. Resistin was injected into LV at the first stage, and AP and HR were evaluated. After that, the paraventricular, supraoptic, and dorsomedial nuclei of the hypothalamus were chosen to evaluate the gene expression of c-Fos. Afterward, resistin was injected into PVN, and cardiovascular responses were monitored. Then to detect possible neural mechanisms of resistin action, agonists or antagonists of glutamatergic, GABAergic, cholinergic, and aminergic transmissions were injected into RVLM.

**Results:**

Resistin injection into LV or PVN could increase AP and HR compared to the control group and before injection. Resistin injection into LV also increases the activity of RVLM, paraventricular, supraoptic, and dorsomedial areas. Moreover, the CV reflex created by the administration of resistin in PVN is probably mediated by glutamatergic transmission within RVLM.

**Conclusion:**

It can be concluded that hypothalamic nuclei, including paraventricular, are important central areas for resistin actions, and glutamatergic transmission in RVLM may be one of the therapeutic targets for high AP in obese people or with metabolic syndrome.

## Introduction

Sympatho excitatory neurons within the rostral ventrolateral medulla (RVLM) are responsible for the tonic and reflex control of the CV response through the production of sympathetic vasomotor tone. Their function is modulated by the supramedullary nuclei, especially the paraventricular nucleus (PVN) (1-3). This nucleus has several types of nerve cells that mediate physiological responses to energy changes (4), environmental stress, and CV diseases (3). Accordingly, PVN is a potential therapeutic target for many autonomic disorders, including metabolic syndrome and cardiac failure (3, 5, 6). The cardiovascular activity of PVN is performed by synthesizing, storing, and releasing vasopressin via axonal projecting to the RVLM and the spinal intermediolateral cell column (IML)(7). PVN axonal projections to the RVLM are seven times greater than axonal projections to the IML (8). Several neurotransmission pathways participate in the generation of basal sympathetic tone to produce the pressor responses in the RVLM, some of which originate from PVN (2, 9). It seems that they can be a treatment target for chronic sympathetic overactivity disorders such as cardiac failure and hypertension (3). 

Resistin is primarily secreted from adipose tissues in rodents (10), macrophages, and monocytes in humans (11, 12). Its expression is affected by gender, age, and diet (13), and its plasma levels are reduced by peroxisome proliferator-activated receptor (PPAR) gamma agonists and increased in dietary and genetic obesity (10). In addition, its expression is regulated by insulin, β-adrenergic agonists, testosterone (14), and tumor necrosis factor (TNF)-α (15). In addition, resistin has a high plasma level in metabolic syndrome, obesity, and CV disorders such as hypertension and HF (16-18). In these conditions, the activity of the sympathetic nerve increase (19, 20), an observation consistent with a resistin‐like molecule, FIZZ 1, which modulates sympathetic neuron functions (21). 

Although it is not clear which regions of the brain are expressed receptors of resistin, the Toll-like receptor 4 (TLR-4) as the receptor of resistin is expressed in the hypothalamus (22, 23). Moreover, resistin can easily pass the blood-brain barrier (24) and exist in cerebrospinal fluid (25). It is widely expressed in the pituitary (13) and arcuate and ventromedial nuclei of the hypothalamus (26), which regulate energy homeostasis and food intake (27). Although many studies have shown a central role for resistin in regulating food consumption, energy homeostasis, and hepatic insulin resistance (28, 29), a few studies have focused on the central cardiovascular function and its treatment sites. Recently, in a pilot study to identify the CV reflex of resistin and determine its optimal dose, we reported that administration of resistin (1, 3, and 5 μg/rat) into the PVN increased AP and HR (30). Furthermore, we demonstrated that neurotransmitters of corticotrophin-releasing factor (CRF) might also be involved in the resistin induced-cardiovascular responses (31). This finding suggested that resistin could produce CV responses, and PVN may be a central site for resistin actions. However, it is not clear if other hypothalamic nuclei can produce the CV responses and whether the response generated by resistin is mediated by direct projections to sympathetic preganglionic neurons within IML or by collateral projections to sympathoexcitatory nerve cells within the RVLM. Nowadays, a wide variety of techniques, including electrophysiology and molecular markers such as c-Fos (32), are used to study the effects of chemical or electrical stimuli on the excitability and activity of nerve cells in different brain regions. The proto-oncogene c-Fos belongs to the immediate-early genes family (33) that is expressed in neurons in response to stimulations (34). Therefore, the first phase of this study aimed to determine the central sites of resistin action by evaluating c-Fos expression following resistin injection into the lateral ventricle. The second phase aimed to find the neurotransmission pathway of resistin-induced CV responses in the parvocellular portion of PVN with RVLM sympathoexcitatory neurons. 

## Materials and Methods

***General procedures***

All steps of this research study were approved by the Ethics Committee of Shiraz University (reference number: 45/4689). Healthy adult male Wistar rats (290-310 g, aged 14-16 weeks) were kept under controlled conditions in terms of light, temperature, and humidity. They had access to chaw and water freely.

***General surgical preparation and cardiovascular response***

Following induction of anesthesia with urethane (1.4 g/kg, IP), arterial pressure was continuously monitored by inserting a heparin-filled polyethylene catheter (PE-50) connected to a pressure transducer (7D polygraph, Grass Instrument Co. USA) into the left femoral artery. Stereotaxic surgery was performed for injecting different substances into LV, PVN, and RVLM, as previously reported (35). Heart rate was continuously recorded by the method described in previous studies (31).

***Experimental protocols***

This study was carried out in two stages. The first stage was designed to survey the effect of resistin injection into the lateral ventricle on AP and HR and to discover the hypothalamic regions which regulate CV response by evaluation of the c-Fos expression. The study groups are as follows:

Group 1: Control group; injection of normal saline into LV (n=8); all of the procedures were the same as the experimental groups; however, instead of resistin, normal saline was injected.

Group 2: Experimental group; resistin (5 µg/rat, Phoenix Pharmaceuticals Inc., Karlsruhe, Germany) was injected into LV.

Resistin and normal saline (NS) were Injected unilaterally into the left side of LV. The volume of injections was the same for all of treatments (5 µl). 

In the second stage, the effects of resistin injection (3 µg/rat) into PVN parvocellular neurons on the CV reflex and its neural transmission within RVLM were evaluated.

Animals were randomly divided into ten groups (n=8) and treated as follows:

1) Control: the vehicle (NS, 1 µl/rat) was injected in the PVN or RVLM.

2) Test 1: received resistin (3 µg/rat) in the PVN and NS in the RVLM through injection.

3) Test 2: received resistin (3 µg/rat) in the PVN and bicuculline (250 nM/rat) in RVLM through injection. 

4) Test 3: received NS in the PVN and bicuculline (250 nM/rat) in RVLM through injection. 

5) Test 4: received NS in the PVN and muscimol (250 nM/rat) in RVLM through injection.

6) Test 5: received resistin in PVN and scopolamine, a muscarinic antagonist (0.3 µM/rat), in the RVLM through injection. 

7) Test 6: received NS in the PVN and scopolamine (0.3 µM/rat) in RVLM through injection. 

8) Test 7: received NS in the PVN, and acetylcholine (0.01 µM/rat) in RVLM through injection.

9) Test 8: received resistin in the PVN and AP5, a selective NMDA receptor antagonist (50 nM/rat), in RVLM through injection.

10) Test 9: received NS in the PVN, and AP5 NMDA receptor antagonist (50 nM/rat) in RVLM through injection. 

11) Test 10: received NS in the PVN and and L-glutamate (50 nM/rat) in RVLM through injection. 

12) Test 11: received resistin in the PV and prazosin, a selective α_1_adrenergic receptor antagonist (10 nM/rat), into RVLM through injection. 

13) Test 12: received Ns in the PVN and prazosin, a selective α_1_adrenergic receptor antagonist (10 nM/rat), into RVLM through injection.

14) Test 13: received NS in the PVN and norepinephrine (50 µM/rat) into RVLM through injection.

***Injection of drugs***

Normal saline, resistin, and other drugs (Sigma-Aldrich) were unilaterally injected into LV, PVN, and RVLM using a 5 μl Hamilton syringe (KH7001, USA). 

***Histological verification***

**Figure 1:**
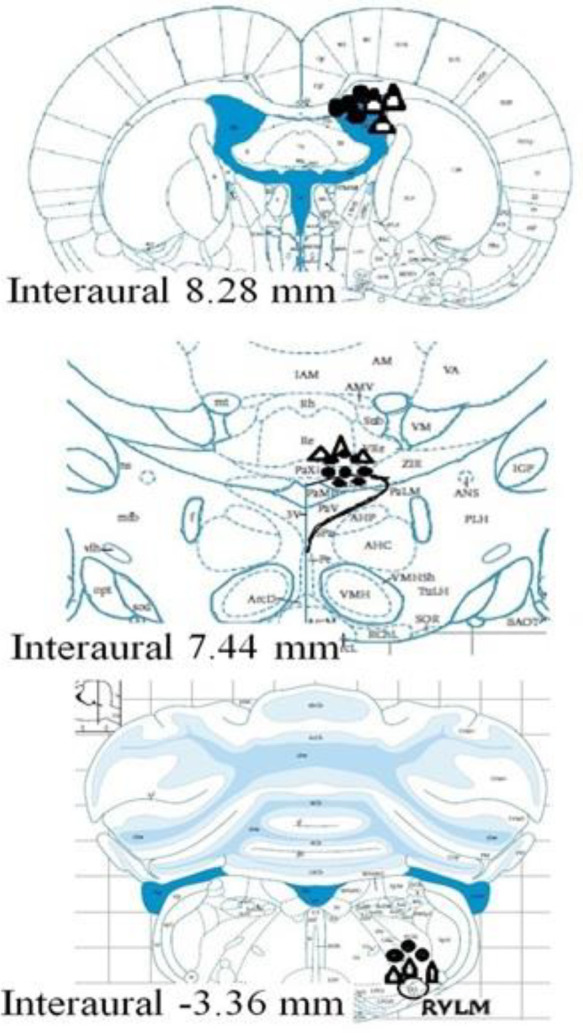
Schematic view of injection site distribution in LV, PVN, and RVLM

Injection sites were confirmed by light microscopy as described in previous studies (31, 35)(Figure 1). Only after confirming the injection site, the obtained cardiovascular data were used for statistical analysis.

***Evaluation of c-Fos and Toll-like Receptor 4 Genes Expression in Different Brain Areas***

The areas of the hypothalamus, including paraventricular, supraoptic, dorsomedial, ventromedial, and RVLM, were selected to evaluate gene expression of c-Fos and toll-like receptor 4 (TLR4). The location of these areas was determined using the coordination in the rat brain atlas (35) and the injection site coordinates, i.e., lateral ventricle coordinates; these sites were obtained by surgical procedure. We also used images and anatomical features from Hahn and Swanson’s (2012) study (36). Total RNA isolation was performed using an RNeasy Mini Kit (Qiagen, Hilden, Germany). Synthesis of cDNA was done by a Prime ScriptTM-RT reagent kit (TaKaRa, Japan). mRNA expression of c-Fos, TLR4, and 18S (internal control) genes (Table 1) was quantified using an SYBR^®^ Green PCR Kit (Qiagen, Hilden, Germany) by a real-time PCR device (Roto- Gene 6000, Corbett, Australia). The quantification of the target genes (c-Fos and TLR4) against the control gene (18S ribosomal) was calculated using the formula 2 ^−ΔΔCT^.

***Data analysis***

The mean level of blood pressure and heart rate were determined 20 min before and 90-120 min after each injection. Baseline levels for each variable were their average levels for 20 min before each injection. Statistical analysis was performed for each variable 20 min before and 90-120 min post-administration in LV, PVN, or RVLM, using repeated measures. The data of gene expression were analyzed using one-way ANOVA followed by *post hoc* Tukey’s test. All results are reported as the mean± standard error of the mean (M±SEM).

## Results

***Gene expression results***

**Figure 2:**
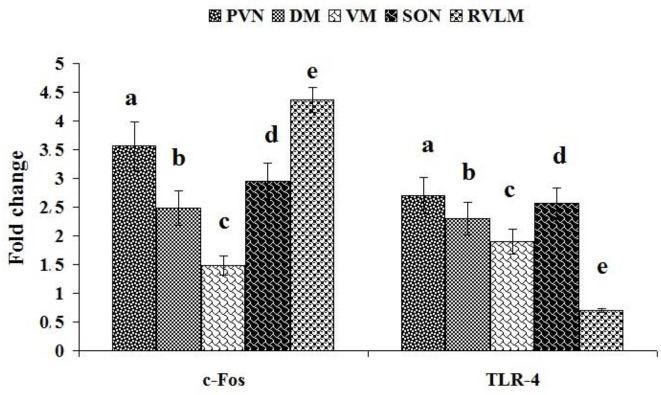
Effect of injection of resistin/saline into LV on the relative expression of c-Fos and Toll-like receptor 4 (TLR4) mRNA in RVLM and different areas of the rat hypothalamus

We have evaluated TLR4 gene expression as a resistin receptor and c-Fos as an indirect marker for neuronal activity in PVN, supraoptic nucleus (SON), ventrolateral (VM), and dorsomedial (DM) of the hypothalamus and RVLM. Comparison of TLR4 gene expression in different areas of the hypothalamus and RVLM, after resistin injection (5 μg/rat) into LV, showed that the highest expression was observed in PVN, SON, VM, and DM.areas, respectively and the lowest in RVLM. Moreover, the highest expression of c-Fos after resistin injection (5 μg/rat) into LV was observed in RVLM, PVN, SON, and DM, respectively, and the lowest in VM (Figure 2). 

***Injection of resistin into the lateral ventricle produced significant cardiovascular responses***

**Figure 3:**
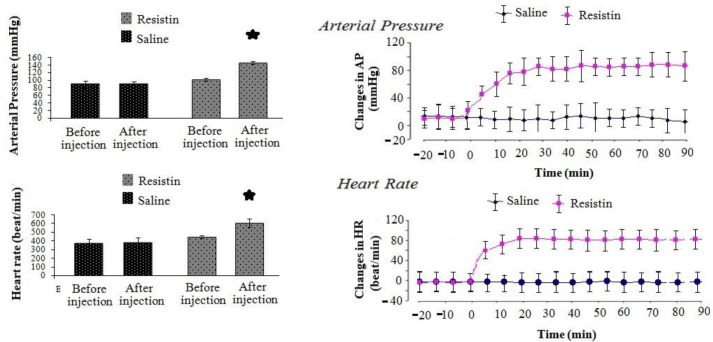
Time course of changes in AP and HR after injection of resistin and NS in LV of anesthetized rats

The mean values (±SEM) of AP and HR pre and post-injection of resistin or NS into LV are demonstrated in Figure 3. Baseline levels of AP and HR in the resistin injection group were 93.12±4.23 mmHg and 375.8±17.5 beats/minute, respectively, while these values in the control group were 92.56±4.14 mmHg and 378.47±14.75 beat/minute (Figure 3). After administration of resistin (5 µg/rat) in LV, AP (F _1, 14_=6.478, *P*<0.05) and HR (F _1, 14_=3.127, *P*<0.05) significantly increased in the test group compared to pre-injection and the control group (Figure 3). A short time after the injection of resistin into LV, AP reached 198±18 mmHg and was unchanged over 90 min. It was also greater than the baseline level of AP (93.12±4.23 mmHg, *P*<0.05) and compared to the control group (92.56±4.14 mmHg, *P*<0.05). Administration of normal saline in the control group did not alter AP and HR compared to pre-injection (*P*>0.05).

***Effect of resistin or NS injection in PVN and agonists or antagonists injection in RVLM on CV response to detecting PVN and RVLM neural connection***

*Cardiovascular response to NS (1 µl/rat) injection into PVN and RVLM: Vehicle group*

**Figure 4:**
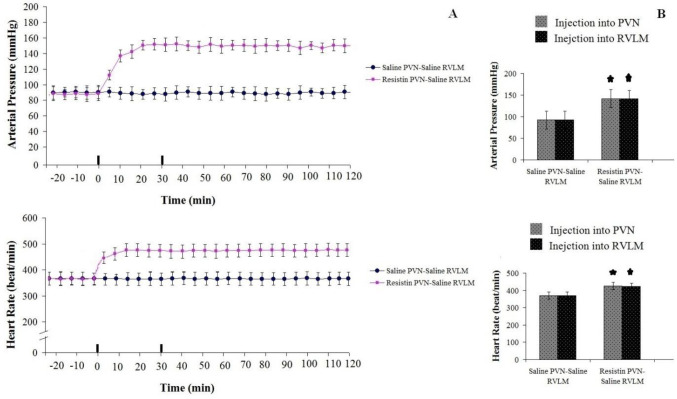
Resistin (3 µg/ rat) injection into PVN increased AP and HR in urethane anesthetized rats

Injection of NS (1 µl/rat) into PVN had no significant change in AP (93.5±7 mmHg) or HR (377±14 beat/min) compared to pre-injection and to the control group. In addition, NS injection (1 µl/rat) in RVLM following its injection PVN did not show a CV response compared to pre-injection (Figure 4).

*Cardiovascular response to resistin (3 µg/rat) injection into PVN and NS (1 µl/rat) injection into RVLM: Test group1*

Baseline values of AP and HR were 96.5±16.4 mmHg and 365.5±45.5 beats/min, respectively. Injection of resistin in the PVN increased AP (ΔAP=+58.5±8.5 mmHg) (F _13, 98_=3.642, *P*<0.05) or HR (ΔHR=+84.5±25.5 beats/min; n=8 rats) (F _13, 98_=16.642, *P*<0.05) compared to pre-injection and to the control group. However, saline administration in the RVLM following resistin injection in the PVN could not change HR and AP compared to before its injection (Figure 4). 

*Cardiovascular response to resistin (3 µg/rat) injection into PVN and bicuculline (250 nM/rat) injected into RVLM: Test group2*

**Figure 5:**
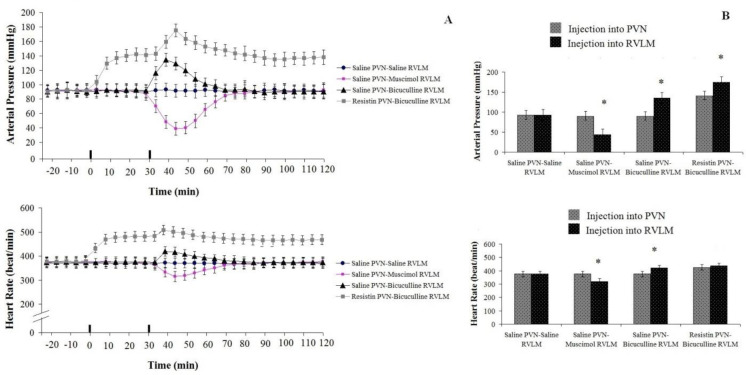
Cardiovascular responses to saline (1 µl/rat) or resistin (3 µg/rat) injection in the PVN and muscimol (250 nM/rat) or bicuculline (250 nM/rat) administration in the RVLM in anesthetized rats

Baseline values of AP and HR were 93±7 mmHg and 374±12 beat/min. Resistin administration (3 µg/rat) in the PVN increased the arterial pressure (ΔAP=+53±8 mm Hg) and the heart rate (ΔHR=76±12beats/min; n=8 rats) compared to pre-injection and to the control group. Bicuculline injection (250 nM/rat) into RVLM following injection of resistin into PVN increased AP (F _13, 98_=16.461, *P*<0.05) while it had no significant effect on HR (F _13, 98_=8.648, *P*>0.05) compared to pre-injection and the control group (Figure 5). 

*Cardiovascular response to injection of NS (1 µl/rat) into PVN and bicuculline (250 nM/rat) into RVLM: Test group 3*

No significant change was observed after NS injection (1 µl/rat) into PVN on AP (90±15 mmHg) and HR (365±21 beat/min) (*P*>0.05)(Figure 5) compared to before injection. While bicuculline injection (250 nM/rat) into RVLM increased AP (ΔAP=+33±10 mm Hg) (F _13, 98_=14.214, *P*<0.05) and HR (ΔHR=+38±12 beats/min; n=8 rats) (F _13, 98_=16.791, *P*<0.05) (Figure 5). 

*Cardiovascular response to injection of normal saline (1 µl/rat) into PVN and muscimol (250 nM/rat) in the RVLM: test group4*

There was no significant change in AP (92.5±11 mmHg) and HR 378±15 (beat/min) after saline (1 µl/rat) injection into PVN compared to pre-injection and the control group. While muscimol administration in the RVLM after saline injection into PVN decreased AP (ΔAP=-53±10 mm Hg) (F _13, 98_=20.364, *P*<0.05) and HR (ΔHR=-68±18 beats/min; n=8 rats) (F _13, 98_=27.597, *P*<0.05) compared to pre-injection (Figure 5). 

*Cardiovascular response to resistin injection (3 µg/rat) in the PVN and scopolamine (0.3 nM/rat) into RVLM: Test group5*

**Figure 6:**
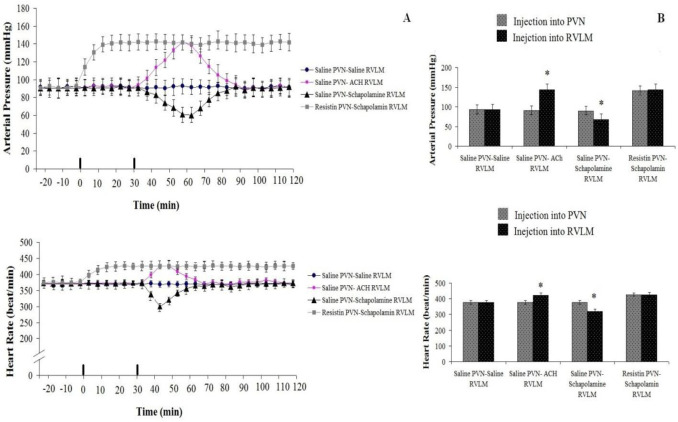
Cardiovascular responses to saline (1 µl/rat) or resistin (3 µg/rat) injection in the PVN and NS, scopolamine (0.3 µM/rat) or acetylcholine (0.01 µM/rat) injection in the RVLM in anesthetized rats

Baseline values of AP and HR were 89.5±10.5 mmHg and 368±25 beat/min. Resistin (3 µg/rat) injection into PVN significantly increased arterial pressure (ΔAP=+63.±18 mm Hg) (F _13, 98_=15.648, *P*<0.05) and heart rate (ΔHR=+74.5±17.5beats/min; n=8 rats) (F _13, 98_=12.541, *P*<0.05) compared to pre-injection and the control group. However, scopolamine (0.3 nM /rat) injection in the RVLM following administration of resistin in the PVN did not show significant changes in AP and HR compared to pre-injection (*P*>0.05, Figure 6). 

*Cardiovascular response to injection of NS (1 µl/rat) into PVN and scopolamine (0.3 nM/rat) into RVLM: Test group 6*

There was no significant change in AP (92±7 mmHg) and HR (364±11 beat/min) post-saline (1 µl/rat) injection into PVN compared to pre-injection and the control group. While, scopolamine (0.3 nM/rat) injection into RVLM after saline into PVN significantly decreased arterial pressure (ΔAP=-33±11 mm Hg) (F _13, 98_=21.361, *P*<0.05) and HR (ΔHR=-23±8beats/min; n=8 rats) (F _13, 98_=5.545, *P*<0.05) compared to pre-injection (Figure 6). 

*Cardiovascular response to injection of NS (1 µl/rat) into PVN and acetylcholine (10* ^-5^ *mM/rat) into RVLM: Test group 7*

There was no significant change in AP (93±6 mmHg) and HR (366±13 beat/min) after saline (1 µl/rat) injection into PVN compared to pre-injection and the control group. While, acetylcholine (10^-5 ^mM/rat) injection in RVLM following NS(1 µl/rat) injection in the PVN could significantly increase AP (ΔAP=+63±10 mm Hg) (F _13, 98_=5.758, *P*<0.05) and HR (ΔHR=+62±11beats/min; n=8 rats) (F _13, 98_=8.584, *P*<0.05) compared to pre-injection (Figure 6). 

*Cardiovascular response to injection of resistin (3 µg/rat) into PVN and AP5 (50 * nM/rat*) in the RVLM: Test group 8 *

**Figure 7:**
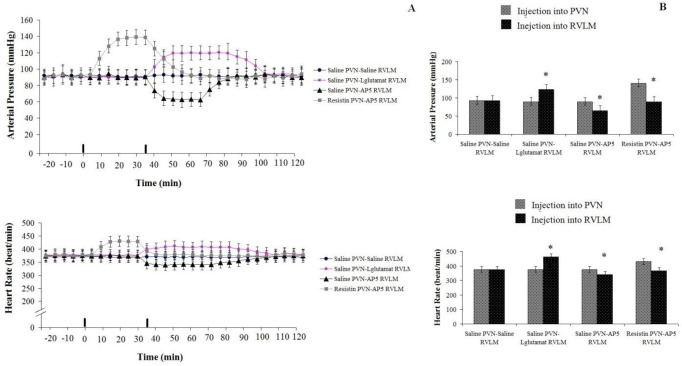
Cardiovascular responses to saline (1 µl/rat) or resistin (3 µg/rat) injection into PVN and saline, AP5 (50 nM/rat) or L-glutamate (50 nM/rat) in the RVLM in urethane anesthetized rats

Resistin (3 µg/rat) injection in the PVN could significantly increase AP and HR compared to pre-injection and the control group. However, administration of AP5 (50 nM/rat) in the RVLM following resistin injection into PVN could significantly decrease AP (ΔAP=-61±10 mm Hg) (F _13, 98_=19.746, *P*<0.05) and HR(ΔHR=-62±11beats/min; n=8 rats) (F _13, 98_=8.961, *P*<0.05) comparing to pre-injection and the control group (Figure 7).

*Cardiovascular response to normal saline (1 µl/rat) injection into PVN and AP5 (50 nM, 1 µl) injection into RVLM: Test group 9*

Baseline values of AP and HR were 90±11 mmHg and 366±15 beat/min. AP5 injection into RVLM following saline injection (1 µl/rat) in the PVN significantly decreased AP (ΔAP=-43±12 mm Hg) (F _13, 98_=12.926, *P*<0.05) and HR (ΔHR=-37±15 beats/min; n=8 rats) (F _13, 98_=10.759, *P*<0.05) compared to pre-injection and to the control group (Figure 7). 

*Cardiovascular response to injection of saline (1 µl/rat) into PVN and L-glutamate (50 nM, 1 µl) in the RVLM: Test group10*

There was no significant change in AP (93±6 mmHg) and HR (366±13 beat/min) after saline (1 µl/rat) injection into PVN compared to pre-injection and the control group. However, L-glutamate injection in the RVLM following NS (1 µl/rat) injection into PVN significantly increased AP(ΔAP=+58.4±14 mm Hg) (F _13, 98_=17.310, *P*<0.05) and HR (ΔHR=+75.5±23beats/min) (F _13, 98_=6.364, *P*<0.05) compared to pre-injection (Figure 7). 

*Cardiovascular response to prazosin (3 mM/rat) injection in RVLM following resistin (3 µg/rat) injection into PVN: Test group 11*

**Figure 8:**
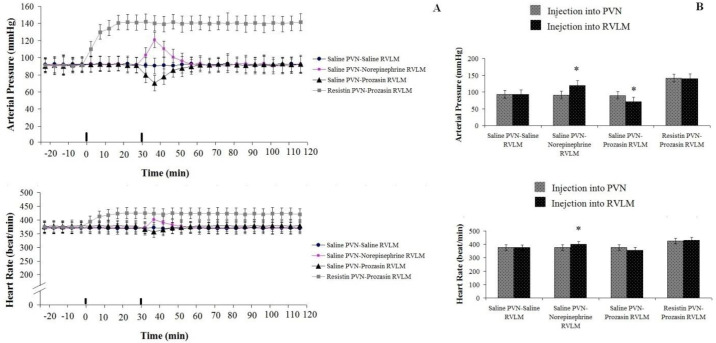
Cardiovascular responses to NS (1 µl/rat) or resistin (3 µg/rat) administration in the PVN and norepinephrine (50 µM/rat) or prazosin (10 nM/rat) injection in the RVLM in anesthetized rats

Resistin (3 µg/rat) injection into PVN significantly increased arterial pressure (ΔAP=+67±14 mm Hg) and heart rate (ΔHR=+75.5±23 beats/min; n=8 rats) compared to pre-injection and to the control group. However, prazosin injection (3 mM/rat) into RVLM following resistin injection (3 µg/rat) into PVN had no significant effects on AP and HR compared to pre-injection (*P*>0.05, Figure 8). 

*Cardiovascular response to injection of prazosin (3mM/rat) into RVLM following saline (1 µl/rat) into PVN: Test group12*

There was no significant change in AP (94±4 mmHg) and HR (361±12 beat/min) after saline (1 µl/rat) injection into PVN compared to pre-injection and the control group. Our data also showed that prazosin (3 mM/rat) administration in RVLM following NS (1 µl/rat) injection into PVN decreased AP (ΔAP=-13±4 mm Hg) and HR (ΔHR=-17±5beats/min; n=8 rats) significantly compared to before injection (F _13, 98_=23.539, *P*<0.05) and (F _13, 98_=6.185, *P*<0.05), respectively (Figure 8). 

*Cardiovascular response to norepinephrine (50 nM /rat) injection in RVLM following saline (1 µl/rat) injection into PVN: Test group 13*

There was no significant change in AP (94±2 mmHg) and HR (368±10 beat/min) after saline (1 µl/rat) injection into PVN compared to before its administration and to the control group. The injection of norepinephrine into RVLM following NS injection (1 µl/rat) into PVN significantly increased AP (ΔAP=28±6 mm Hg) (F _13, 98_=24.352, *P*<0.05) and HR (ΔHR=45.5±8 beats/min; n=8 rats) (F _13, 98_=3.798, *P*<0.05) comparing with pre-injection and to the control group (Figure 8). 

## Discussion

Resistin (5 µg/rat) injection into the lateral ventricle could increase c-Fos expression in paraventricular, supraoptic, and dorsomedial areas of the hypothalamus and RVLM, which was also supported by two other studies (28, 34). However, its expression had no significant change in the ventromedial of the hypothalamus. Previous studies by the immunohistochemistry (IHC) of FOS protein showed that the arcuate nucleus (ARN) of the hypothalamus may be one of the central areas of resistin action (26). Singhal *et al*. (2007) reported that central resistin injection (6 µg/mice) increases the FOS protein disruption in the dorsomedial, PVN, and hypothalamic arcuate nuclei (28). Another study showed that resistin (7 µg/rat) administration in LV enhances FOS protein expression in SON, PVN, and sub-fornical organs (SFO) but not in ARN and DM (34). The inconsistency of the results could be caused by different doses of injectable resistin, sampling time, and other protocols study. Our results also indicated that c-Fos was also expressed at high levels in RVLM. This result indicates that RVLM, as a main controlling area of cardiovascular activity, can be activated by resistin and produce cardiovascular responses. 

The results showed that TLR-4 as the resistin receptor was expressed in PVN, SON, VM, and DM of the hypothalamus as previously reported (22, 28); while in RVLM, its expression was very low. Benomar *et al*. (2013) showed that TLR4, as a resistin receptor, is expressed in the hypothalamus and mediates the actions of resistin (22). They showed that central resistin through TLR4 and activating the proinflammatory pathways induces insulin resistance. In addition, it has been shown that resistin injection in LV enhances sympathetic nerve activity (SNA) (37) via activating the phosphatidylinositol 3-kinase (24). Based on the results of the expression of these genes (TLR4 and c-Fos), it can be suggested that resistin has receptors in different regions of the hypothalamus. Although we in this study only evaluated the cardiovascular activity of resistin, other studies have suggested several crucial roles for it in the hypothalamus, such as regulation of hypothalamic and peripheral lipid metabolism (27), appetite (26), and sympathetic nerve activity(34, 38).

Our results showed that the time course of CV response to injection of resistin (5 µg/rat) in the LV was very quick and long-lasting. The rapid CV response to resistin actions likely reflects the time course of activation of intracellular signaling pathway components (39, 40). We have also shown that resistin, in addition to paraventricular and dorsomedial areas, can activate the supraoptic area. Stimulation of these areas by resistin may increase the release of vasopressin, which can cause a long-term cardiovascular response. However, these hypotheses need to be examined. It is possible that injected resistin into the LV, in addition to the hypothalamic nuclei, produces the cardiovascular response in other nuclei that have cardiovascular activity through cerebrospinal fluid circulation. We also showed that the administration of resistin into LV increases AP and HR, which was inconsistent with others (34, 41). Kosari *et al*. (2011) indicated that resistin injection (7 µg/rat) into the lateral ventricle (1) promoted the activity of the sympathetic nerve and decreased its activity to brown adipose tissue, (2) increased c-Fos protein expression in SON, magnocellular, and the parvocellular portions of PVN and SFO, but not in ARN and DM and (3) did not affect mean AP and heart rate (34). Direct relationships between plasma levels of resistin and cardiovascular diseases have already been reported (16-18, 42). Increased distribution of c-Foc protein, which is known as a biomarker of nerve cell activity, in response to injection of resistin into LV has been reported in parvocellular and magnocellular subdivisions of the PVN (34). Since the function of the cardiovascular system is controlled by PVN, especially the parvocellular area (2, 3), the obtained results in our study are rational. In a preliminary study to investigate the cardiovascular effects of resistin and to determine the appropriate dosage for the cardiovascular response, we indicated that resistin (1, 3, and 5 µg/rat, 1 µl) injection into PVN increases AP, HR, and the height of QRS complex (30). 

Chemical stimulation of the PVN by L-glutamate (43, 44) decreases BP, HR, and renal SNA in anesthetized rats via NMDA glutamate receptors (45). Stimulation of glutamate receptors causes nitric oxide (NO) production which enhances GABA inhibitory effects in the PVN (3, 8). Moreover, a study suggested that parvocellular neurons of PVN receive excitatory input from norepinephrine-sensitive local glutamatergic interneurons (46). We showed that TLR4 as the receptor of resistin and c-Fos as an indirect factor for neural activity is highly expressed in PVN, SON, and DM. These results are consistent with previous studies (22, 28, 34, 38). We know that central resistin is highly expressed in some hypothalamus and pituitary nuclei (27, 28, 47) and peripheral resistin, which is expressed by macrophages and adipocytes, can easily pass through the BBB (24) and reach the regions of actions including the hypothalamus. In addition to this description, a study indicated that resistin can inhibit the secretion of norepinephrine and dopamine in the hypothalamus (48). Therefore, resistin produced CV responses probably by affecting the interaction between glutamatergic and noradrenergic synapses inhibiting GABAergic inputs that have an essential effect in controlling PVN activity and sympathetic outflow. However, it needs to be investigated. Moreover, the intracellular mechanisms of resistin in PVN paracellular neurons are not fully understood. However, Benomar *et al*. (2013) reported for the first time that TLR4 acts as the site for resistin in SH-SY5Y human neuroblastoma cells and the hypothalamus (22). Other researchers also indicated that resistin in pituitary somatotrope cells could activate (1) the Gs protein-dependent mechanism, (2)the Adenylate cyclase /cAMP/protein kinase A pathway, (3) the phosphatidyl inositol 3-kinase/Akt pathway, and (4) protein kinase C and extracellular Ca^2+^ influx through L-type Ca^2+^ channels(39). The cardiovascular response induced by resistin injected into the PVN may be modulated via (i) direct projections to sympathetic preganglionic neurons in the IML (49-51) or (ii) unilateral projections from parvocellular neurons of PVN to sympathoexcitatory neurons of the RVLM (8, 10, 28, 52). The next step of the research aimed to determine the neuronal connection of the PVN with the RVLM that mediates the generated cardiovascular response to resistin, and the dose of 3 μg/rat of resistin was selected. In this step, resistin or normal saline was injected into PVN to produce or not produce a cardiovascular response, respectively, then agonists or antagonists of cholinergic, GABAergic, catecholaminergic, and glutamatergic systems were injected into the RVLM. Resistin injection into PVN and saline into RVLM could induce a fast and powerful cardiovascular response which reflects the time course of the activation of the intracellular signaling pathways (39, 40). Moreover, this response may be due to potentiating and inhibiting inputs to PVN. The results showed that muscimol injection into RVLM following saline injection into PVN decreased AP and HR, which is in agreement with reports by other researchers (53, 54). Moreover, the severity of the cardiovascular reflex produced by bicuculline injection in the RVLM following resistin into PVN was similar to the finding reported by Horiuchi and Dampney (2003) (53) and Peng *et al*. (2002) (55). It was reported that administration of an agonist GABA_A_ and muscimol in the PVN (30, 56) or RVLM (57) inhibits renal sympathetic nerve activity, AP, and HR within seconds. It’s well understood that “the caudal ventrolateral medulla has GABAergic neurons that induce a tonic inhibitory effect on the RVLM and relay the inhibitory inputs from the NTS” (2, 3). Hence, it is reasonable that injection of antagonist and agonist of GABA receptor in this area causes pressor or depressor response, respectively. In addition, Ach (10^-5^ mM) injection into RVLM following saline into PVN increased cardiovascular response, which is in agreement with another study (1997 and 2000) (58, 59). Moreover, the cardiovascular response generated by resistin injection in the PVN was not inhibited by scopolamine injection into RVLM, which disagrees with a report by Kubo *et al*. (2000)(58). Kubo *et al*. (2000) suggested that the chemical and electrical stimulations of PVN could activate cholinergic inputs from the lateral parabrachial nucleus (LPBN) to the RVLM and increase AP. Anatomical evaluations showed that some PVN neurons project to the LPBN (58), and chemical and electrical PVN stimulation increases the function of neurons in the LPBN. They also showed that GABA injection into the LPBN inhibits the pressor response generated by PVN stimulation (58). It can be hypothesized that stimulation of the PVN can activate neurons in the LPBN, which in turn can activate cholinergic inputs to neurons in the RVLM. 

Our findings showed that L-glutamate injection into RVLM increases CV responses, which is in agreement with (2, 51, 60, 61). The results of our study have also shown that AP5 administration in RVLM decreased the CV response produced by resistin injection into PVN, which supports the report that proposed that glutamate as an excitatory neurotransmitter acts through NMDA and AMPA receptors in sympathoexcitatory neurons of the RVLM (61). It can be suggested that glutamatergic projection from the PVN to the sympathoexcitatory neurons of RVLM contributes to the pressure responses. So at first glance, the highlight of this study is that resistin-induced pressor responses are mediated through glutamatergic transmission in the RVLM. The results also showed that the pattern of resistin-induced pressor responses was somewhat similar to the pattern produced by glutamate injection into RVLM. Both BP and HR, after injection, sharply began to rise. Moreover, comparing these findings showed that the resistin-induced pressor responses were significantly stronger and had longer duration than those produced by injection of glutamate in the RVLM. Moreover, angiotensin II (Ang II) and CRF as co-neurotransmitters with glutamate in RVLM generate pressor responses that originate from the PVN (62, 63). In addition, immunocytochemical studies showed that PVN neurons have oxytocin and vasopressin projects to the sympathetic preganglionic neuron and may affect the function of IML of the spinal cord (2, 3). Therefore, it seems that a part of the CV reflex generated by resistin injection could be mediated by neural pathways that do not project to the RVLM, which requires further investigation.

The filled circle shows the injection site of normal saline in LV, PVN, and RVLM, and the open triangle shows the injection site of resistin in LV or PVN. The open pentagon also shows the injection site of the drugs in RVLM

LV: lateral ventricle, PVN: Paraventricular Nucleus, RVLM: Rostral Ventrolateral Medulla

Different alphabetic letters show significant differences between different areas (*P<*0.05, n=8)

PVN: Paraventricular Nucleus, DM: Dorsomedial, VM: Ventromedial, SON: Supraoptic Nucleus, RVLM: Rostral Ventrolateral Medulla, LV: lateral ventricle

**Table 1:** The sequence of the primers of the target and housekeeping genes used in this study

Gene	Sequence	PCR product (bp)
c-Fos	F-5′- AGCATGGGCTCCCCTGTCA-3′ R-5′- GAGACCAGAGTGGGCTGCA-3′	134
Toll-like receptor 4	F- 5′- GCATCATCTTCATTGTCCTTGAGA R-5′- CTCCCACTCGAGGTAGGTGTTT	101
18Sribosomal RNA	F-5′- CCCAGTAAGTGCGGGTCATA-3′ R-5′- GGCCTCACTAAACCATCCAA-3′	96

Zero time in the vertical axis indicates the baseline of AP and HR. Time zero on the horizontal axis represents the injection time into LV. A significant difference between the saline and resistin groups was observed five min after resistin administration and later. The left charts represent the alteration of AP and HR in both groups pre and post-resistin or saline injection. *P<0.05

AP: Arterial Pressure; HR: Heart Rate; LV: lateral ventricle; NS: normal saline

A: Time course of changes in AP and HR after NS or resistin injection into PVN and NS injection into RVLM. Time zero, or the first vertical line, is NS or resistin injection into PVN. The second vertical line is the injection time into the RVLM. A significant difference was observed five min after injection of resistin and later. B: mean±SEM of AP and HR values pre and post-resistin injection into the PVN and saline into RVLM (n=8). * P<0.05

PVN: paraventricular nucleus; AP: Arterial Pressure; HR: Heart Rate; NS: normal saline; RVLM: rostral ventrolateral medulla

A: Time course of changes in AP and HR to NS or resistin injection into PVN and injection of NS, muscimol, or bicuculline into RVLM. Time zero, or the first vertical line, is saline or resistin injection into PVN. The second vertical line is the injection time into the RVLM. From five min after resistin injection in the PVN and other drugs in the RVLM, a significant difference was observed between groups. B: Mean±SEM of AP and HR values pre and post-NS or resistin injection in the PVN and NS or other drugs into RVLM (n=8). * *P<*0.05

PVN: paraventricular nucleus; AP: Arterial Pressure; NS: normal saline; RVLM: rostral ventrolateral medulla

A: Time course of changes in AP and HR to NS or resistin injection into PVN and injection of saline, acetylcholine, or scopolamine into RVLM. Time zero, or the first vertical line, is saline or resistin injection in the PVN. The second vertical line is the injection time into the RVLM. Five min after resistin injection in the PVN and other drugs in the RVLM a significant difference was observed among different groups. B: Mean±SEM of AP and HR values pre and post-NS or resistin injection into the PVN and NS or drugs into RVLM (n=8). * *P<*0.05

PVN: paraventricular nucleus; AP: Arterial Pressure; NS: normal saline; RVLM: rostral ventrolateral medulla

A: Time course of changes in AP and HR to NS or resistin injection in the PVN and injection of NS, AP5, or L-glutamate in the RVLM. Time zero, or the first vertical line, is saline or resistin injection into PVN. The second vertical line is the injection time into the RVLM. Five min after injection of resistin and later a significant difference was observed between saline, resistin, and other drugs. B: Mean±SEM of AP and HR values pre and post-NS or resistin injection in the PVN and NS or drugs into RVLM (n=8). *P<0.05

PVN: paraventricular nucleus; AP: Arterial Pressure; HR: Heart Rate; NS: normal saline; RVLM: rostral ventrolateral medulla

A: Time course of the response in AP and HR to NS or resistin injection into PVN and injection of NS, norepinephrine (50 µM/rat), or prazosin (10 nM/rat) injection into RVLM. Time zero, or the first vertical line, is NS or resistin injection in the PVN. The second vertical line is the injection time into the RVLM. Five min after administration of resistin into the PVN and other drugs in the RVLM, a significant difference was observed between the different groups. B: Mean±SEM of AP and HR values pre and post-saline or resistin injection into the PVN and NS or drugs into RVLM (n=8). * *P<*0.05

PVN: paraventricular nucleus; AP: Arterial Pressure; HR: Heart Rate; NS: normal saline; RVLM: rostral ventrolateral medulla

## Conclusion

It can be concluded that (I) injection of resistin in LV can induce cardiovascular responses, (II) PVN, DM, and SON may be central sites for actions of resistin, (III) expression of c-Fos in the RVLM increases after the injection of resistin into LV, (IV) Toll-like receptor 4, as a resistin receptor, is highly expressed in PVN, SON, and DM of the hypothalamus. (V) Resistin injection into PVN produces cardiovascular responses, and (VI) its neural transmission within RVLM may be mediated by glutamatergic transmission. Overall, these data suggest that PVN and other nuclei of the hypothalamus activation by resistin increase the glutamatergic drive to RVLM and induce the pressor responses. These results can propose a new approach to the identification of the pathogenesis and treatment of complications of metabolic disorders such as hypertension and CHF.

## Authors’ Contributions

A A and G J designed the experiments; A A performed experiments and collected data; A A, S H, and G J discussed the results and strategy; G J supervised, directed, and managed the study; A A, S H, and G J approved the final version to be published.

## Conflicts of Interest

The authors declare that they have no competing interests.
